# MicroRNA-30d regulates cardiomyocyte pyroptosis by directly targeting foxo3a in diabetic cardiomyopathy

**DOI:** 10.1038/cddis.2014.430

**Published:** 2014-10-23

**Authors:** X Li, N Du, Q Zhang, J Li, X Chen, X Liu, Y Hu, W Qin, N Shen, C Xu, Z Fang, Y Wei, R Wang, Z Du, Y Zhang, Y Lu

**Affiliations:** 1 Department of Pharmacology (State-Province Key Laboratories of Biomedicine-Pharmaceutics of China, Key Laboratory of Cardiovascular Research, Ministry of Education), Harbin Medical University, Harbin 150081, China; 2 Department of General Surgery, The First Affiliated Hospital, Harbin Medical University, Harbin 150001, China; 3 Department of Geriatrics, The Second Affiliated Hospital, Harbin Medical University, Harbin 150081, China; 4 Institute of Clinical Pharmacy, The Second Affiliated Hospital, Harbin Medical University, Harbin 150081, China; 5 Institute of Cardiovascular Research, Harbin Medical University, Harbin 150081, China

## Abstract

Diabetic cardiomyopathy is a common cardiac condition in patients with diabetes mellitus, which can result in cardiac hypertrophy and subsequent heart failure, associated with pyroptosis, the pro-inflammatory programmed cell death. MicroRNAs (miRNAs), small endogenous non-coding RNAs, have been shown to be involved in diabetic cardiomyopathy. However, whether miRNAs regulate pyroptosis in diabetic cardiomyopathy remains unknown. Our study revealed that mir-30d expression was substantially increased in streptozotocin (STZ)-induced diabetic rats and in high-glucose-treated cardiomyocytes as well. Upregulation of mir-30d promoted cardiomyocyte pyroptosis in diabetic cardiomyopathy; conversely, knockdown of mir-30d attenuated it. In an effort to understand the signaling mechanisms underlying the pro-pyroptotic property of mir-30d, we found that forced expression of mir-30d upregulated caspase-1 and pro-inflammatory cytokines IL-1*β* and IL-18. Moreover, mir-30d directly repressed foxo3a expression and its downstream protein, apoptosis repressor with caspase recruitment domain (ARC). Furthermore, silencing ARC by siRNA mimicked the action of mir-30d: upregulating caspase-1 and inducing pyroptosis. These findings promoted us to propose a new signaling pathway leading to cardiomyocyte pyroptosis under hyperglycemic conditions: mir-30d↑→foxo3a↓→ ARC↓→caspase-1↑→IL-1*β*, IL-18↑→pyroptosis↑. Therefore, mir-30d may be a promising therapeutic target for the management of diabetic cardiomyopathy.

Diabetic cardiomyopathy is a leading cardiovascular complication occurring in approximately 60% of patients with well-controlled diabetes.^1^ It frequently occurs when systolic function is impaired in the presence of diastolic dysfunction, independent of any vascular diseases or hypertension.^1^ Accumulating evidence has implicated hyperglycemia, lipotoxicity and mitochondrial uncoupling as contributors to cardiac inflammation, which has an essential role in the onset and development of diabetic cardiomyopathy.^2, 3, 4^

MicroRNAs (miRNAs) are endogenous, small non-coding RNAs of approximately 19–22 nucleotides in length that anneal inexactly to complementary sequences in the 3′-untranslated regions (3′-UTRs) of target mRNAs to either facilitate their degradation or repress the translation process.^5^ Numerous studies have shown that miRNAs are involved in a wide variety of biological processes, including cell proliferation, differentiation, metastasis, apoptosis and immune responses,^6,7^ and also function as prognostic markers in the development and progression of cardiovascular diseases by targeting pertinent genes.^4,5,8, 9, 10^

Pyroptosis is pro-inflammatory programmed cell death.^11,12^ It has biochemical and morphological characteristics of necrosis and apoptosis, but unlike apoptosis or necrosis, pyroptosis results in the release of cytokines that activate pro-inflammatory immune cell mediators.^13^ Caspase-1 is activated during pyroptosis by a large supramolecular complex known as the pyroptosome^14^ and subsequently processes the proforms of interleukin (IL)-1*β* and IL-18, the inflammatory cytokines, into their active forms.^15,16^ However, few studies have focused on the participation of miRNAs in pyroptosis in diabetic cardiomyopathy.

The aim of this study was to elucidate the essential role of miRNAs in regulating diabetic cardiomyopathy and the underlying mechanisms. In this study, we demonstrated that mir-30d promoted cardiomyocyte pyroptosis by directly targeting Forkhead box O3 (Foxo3a), a crucial regulator of diverse cellular activities, such as cell cycle arrest, oxidative scavenging, cell proliferation, survival and death.^17,18^ The downstream protein, apoptosis repressor with caspase recruitment domain (ARC), which antagonizes both the intrinsic and the extrinsic pathways of cell death,^19, 20, 21^ was subsequently inhibited. Taken together, we verified that mir-30d has a crucial role in the pathogenesis of cardiomyocyte pyroptosis, suggesting that mir-30d may be a potential therapeutic target in the treatment of diabetic cardiomyopathy.

## Results

### Cardiac dysfunction in streptozotocin (STZ)-induced diabetic rats

**Figure 1:**
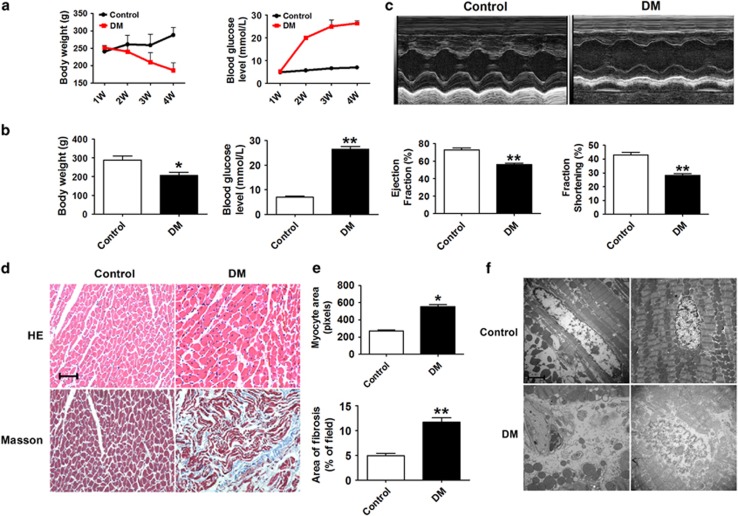
Cardiac function in STZ-induced diabetic rats. (**a**) Body weight and fasting blood glucose levels measured at 4 weeks after STZ injection in the control and DM groups. (**b**) Body weight and blood glucose levels during the last week before rats were anesthetized. *n*=5. (**c**) Echocardiogram and ejection fraction (EF %) and fractional shortening (FS %) measurements of control and DM rats at the end of the fourth week before rats were anesthetized. *n*=3. (**d**) HE staining and Masson staining of cross-sectional tissue slices of heart tissue in the control and DM groups ( × 200). Scale bar: 20 *μ*m. *n*=4. (**e**) Areas of myocyte and fibrosis in control and DM rats. *n*=5. (**f**) Transmission electron microscopy micrograph results in the control and DM groups ( × 10 000). *n*=3. *P<0.05 & **P<0.01 *versus* control; mean±S.E.M.

To study diabetic cardiomyopathy, we first developed a rat model of diabetes mellitus (DM) induced by STZ and high-fat diet.^22,23^ As shown in Figures 1a and b, DM rats showed significant increases in fasting blood glucose levels, blood triglycerides (TG), total cholesterol (TC), low-density lipoprotein (LDL) and water intake, accompanied by decreases in body weight and high-density lipoprotein (HDL) (Supplementary Table S1). These results indicated the DM rats had typical diabetic phenotypes, including hyperglycemia and hyperlipidemia.

As shown in Figure 1c, echocardiography and hemodynamic measurements demonstrated that ejection fraction (EF %) and fractional shortening (FS %) were both remarkably decreased in DM rats compared with those in the control group, indicating impaired cardiac function. Myocardial hypertrophy and interstitial fibrosis are key causes for depressed cardiac function in diabetic hearts. Hematoxylin and eosin (HE) and Masson trichrome staining of cardiac tissues revealed clear structural abnormalities, most notably increases in both myocardial cell size and interstitial fibrotic areas in the DM group compared with the control group (Figures 1d and e). Moreover, transmission electron microscopy images revealed disordered sarcomeres, swollen mitochondria, increased interstitial collagen, loss of intracellular contents and the presence of pyroptosome in the DM hearts, suggesting cardiac dysfunction (Figure 1f).

### High glucose induces cardiomyocyte pyroptosis

**Figure 2:**
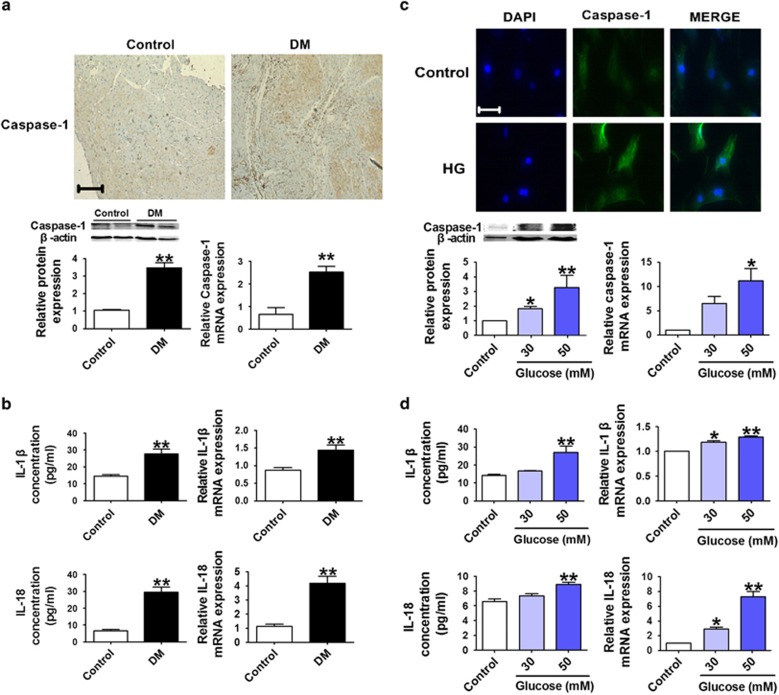
High glucose promotes cardiomyocyte pyroptosis. (**a**) Caspase-1 levels using immunohistochemical ( × 200) staining in control and DM rats. Relative mRNA and protein levels of caspase-1 in the control and DM groups in the bottom panel. (**b**) IL-1*β* and IL-18 concentrations in serum and mRNA expression in the rat hearts. (**c**) Immunofluorescence results ( × 400) indicating the expression of caspase-1 in normal glucose (control) and high-glucose-treated cardiomyocytes (HG). Blue: nuclear staining (DAPI); green: caspase-1 staining (scale bar: 20 *μ*m). Relative protein and mRNA expression of caspase-1 in cardiomyocytes treated with different concentrations of glucose (control, 30 and 50 mM) in the bottom panel. (**d**) IL-1*β* and IL-18 concentrations and mRNA levels in cardiomyocytes treated with different concentrations of glucose (control, 30 and 50 mM). *n*=4. *P<0.05 & **P<0.01 *versus* control; mean±S.E.M.

Immunohistochemical analysis of rat hearts showed that expression of caspase-1 was remarkably increased at both the protein and mRNA levels in the DM group, and similar changes in IL-1*β* and IL-18 were consistently observed (Figures 2a and b). Consistently, in cultured cardiomyocytes incubated with 30 mmol/l or 50 mmol/l glucose (HG) to mimic hyperglycemia, IL-1*β* and IL-18 levels were also found significantly increased, indicating an induction of cardiomyocyte pyroptosis (Figures 2c and d).

### Mir-30d is upregulated and foxo3a is downregulated in hyperglycemic conditions

**Figure 3:**
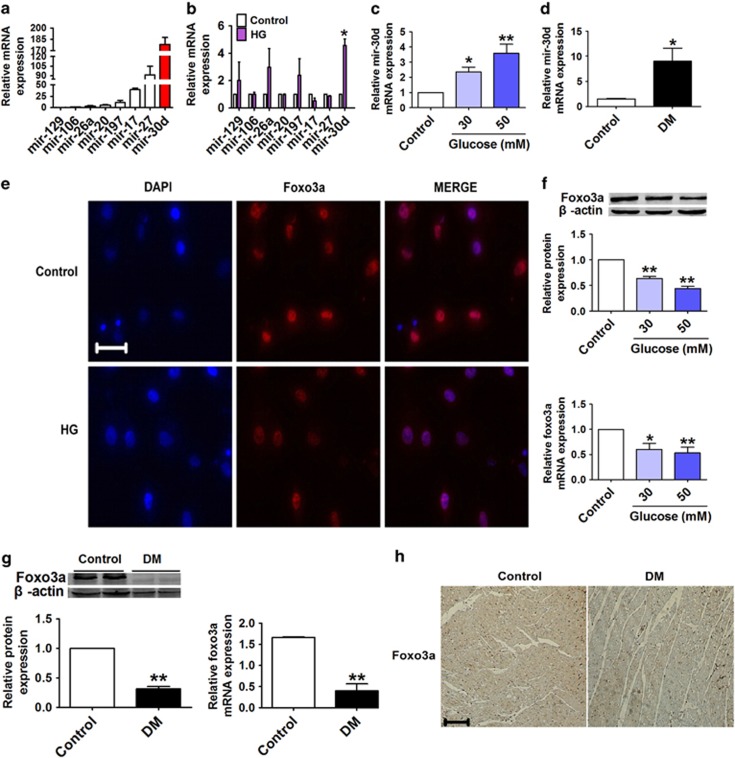
Mir-30d is upregulated and foxo3a is downregulated in high-glucose-treated cardiomyocytes and diabetic rats. (**a** and **b**) Relative mRNA levels of mir-129, mir-106, mir-26a, mir-20, mir-197, mir-17, mir-27 and mir-30d in cardiomyocytes under normal and high-glucose (50 mM) conditions. (**c** and **d**) Mir-30d levels measured by real-time PCR both in cardiomyocytes treated with different concentrations of glucose (control, 30 and 50 mM) and in the hearts of control and DM rats. (**e**) Immunofluorescence results ( × 400) indicating the expression of foxo3a in normal glucose (control) and high-glucose-induced cardiomyocytes. Blue: nuclear staining (DAPI); red: foxo3a staining; violet: merged images. (**f**) Expression of foxo3a at protein and mRNA levels in cardiomyocytes treated with different concentrations of glucose. (**g**) Relative protein and mRNA levels of foxo3a in the hearts of control and DM rats. (**h**) Foxo3a expression of consecutive sections of hearts in control and DM rats assessed by immunohistochemical staining. Scale bar: 20 *μ*m. *n*=3. *P<0.05 & **P<0.01 *versus* control; mean±S.E.M.

We further investigated the possible involvement of miRNAs in high-glucose-treated cardiomyocytes to mimic hyperglycemia. We began by measuring the levels of several miRNAs reportedly associated with cardiovascular diseases, including mir-129, mir-106, mir-26a, mir-20, mir-197, mir-17, mir-27 and mir-30d,^24, 25, 26, 27, 28, 29^ in cardiomyocytes under both normal and high-glucose conditions. The results showed that mir-30d demonstrated the most pronounced deregulation in the high-glucose environment (Figures 3a and b), and high-glucose treatment increased mir-30d expression in a dose-dependent manner (Figure 3c). We subsequently determined whether mir-30d was involved in the pathogenesis of diabetic cardiomyopathy in rats. Overexpression of mir-30d was consistently observed in the DM rats compared with the control animals (Figure 3d). Moreover, we observed a significant downregulation of foxo3a, a transcription factor having a critical role in the regulation of cell death in cardiomyocytes,^30^ in high-glucose-treated cardiomyocytes, as determined by immunofluorescence staining, real-time PCR and western blotting analysis (Figures 3e and f). Similar downregulation of foxo3a was also observed in DM rats (Figures 3g and h).

### Mir-30d directly targets foxo3a

**Figure 4:**
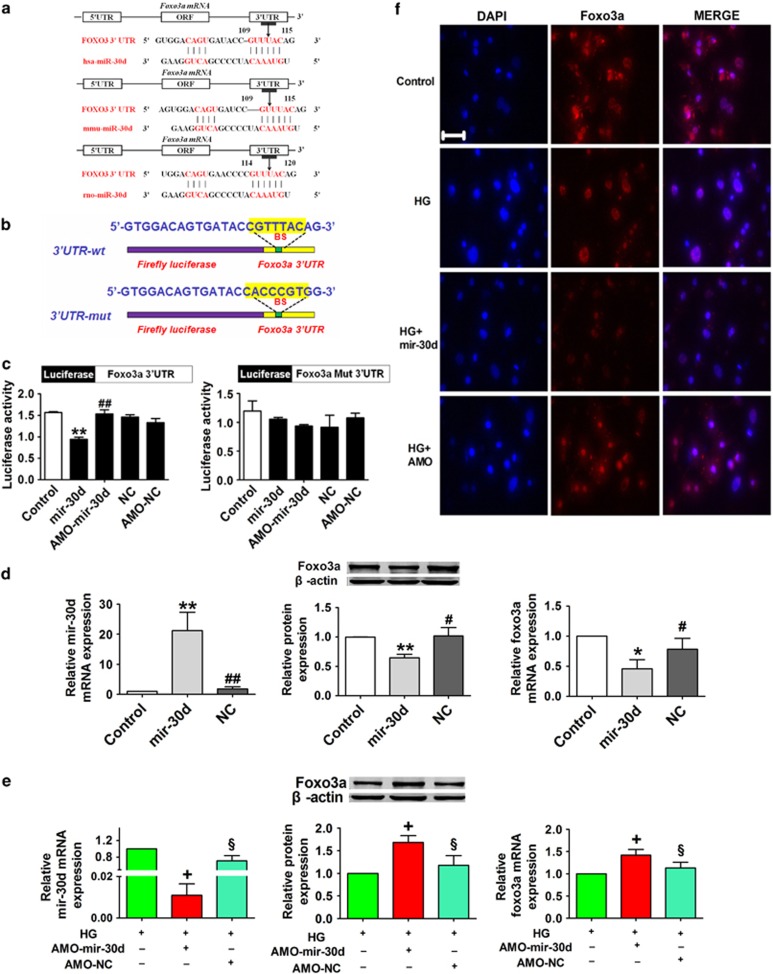
Foxo3a is a direct target of mir-30d. (**a**) Combination sequences of hsa-mir-30d, mmu-mir-30d and rno-mir-30d with the 3′-UTR of foxo3a. (**b**) Foxo3a 3′-UTR construct mutated at the predicted miR-30d site. (**c**) Luciferase reporter activities of chimeric vectors carrying the luciferase gene and a fragment of the foxo3a 3′-UTR containing the binding sites of miR-30d in the control, mir-30d, AMO-mir-30d, NC and AMO-NC groups. (**d**) Levels of mir-30d and foxo3a in the control, mir-30d and NC groups. (**e**) Knockdown of mir-30d resulted in increased foxo3a levels in cardiomyocytes under high-glucose condition. *n*=3. (**f**) Immunofluorescence images showing the location of foxo3a in the control, HG, HG+ mir-30d and HG+AMO-mir-30d groups. Blue: nuclear staining (DAPI); red: foxo3a staining; violet, merged images. Scale bar: 20 *μ*m. *P<0.05 and **P<0.01 *versus* control; ^#^*P*<0.05 and ^##^*P*<0.01 *versus* mir-30d; ^+^*P*<0.05 *versus* HG; ^§^*P*<0.05 *versus* HG+AMO-mir-30d; mean±S.E.M.

We next performed a series of functional studies to determine the link between mir-30d and foxo3a. Computational analysis predicted a conserved binding site for mir-30d in the 3′-UTR of foxo3a gene (Figure 4a). To verify that mir-30d directly targets foxo3a, we prepared luciferase constructs carrying the foxo3a 3′-UTR (Figure 4b). Cotransfection of mir-30d with the luciferase reporter vector into HEK293 cells caused a sharp decrease in luciferase activity compared with transfection of the luciferase vector alone. The mir-30d-induced depression of luciferase activity was rescued by an antisense inhibitor oligonucleotide (AMO-mir-30d) used to knockdown mir-30d. However, mir-30d failed to affect the luciferase activity elicited by the construct carrying the foxo3a 3′-UTR with the mutant mir-30d-binding site (Figure 4c). As depicted in Figures 4d and e, transfection of mir-30d into cultured cardiomyocytes remarkably reduced the protein and mRNA levels of foxo3a. Conversely, foxo3a was significantly upregulated when AMO-mir-30d was transfected into cardiomyocytes under mimicked hyperglycemic conditions (50 mmol/l glucose; HG), indicating that foxo3a is a direct target of mir-30d. Consistently, immunofluorescence staining also demonstrated an approximately 50% decrease in foxo3a level in HG conditions. The abundance of foxo3a expression was decreased remarkably in the HG and HG+mir-30d groups, and this decrease was corrected by AMO-mir-30d transfection (Figure 4f).

### ARC acts as a downstream factor of foxo3a

**Figure 5:**
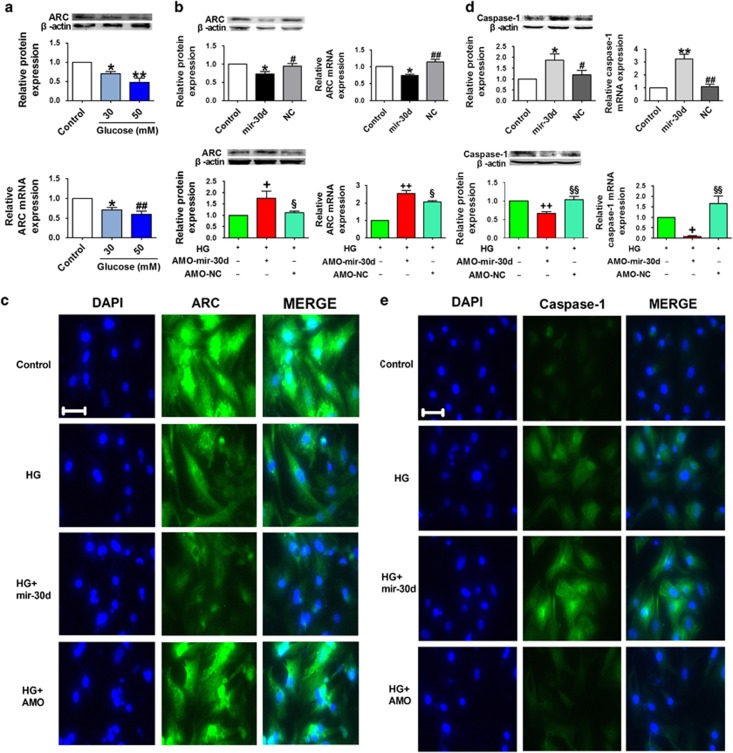
Mir-30d upregulates caspase-1 expression through ARC. (**a**) Relative protein and mRNA levels of ARC in cardiomyocytes treated with different concentrations of glucose (control, 30 and 50 mM). (**b**) Relative protein and mRNA levels of ARC in the control, mir-30d and NC groups. ARC levels in the HG, HG+AMO-mir-30d and HG+AMO-NC groups in high-glucose-treated cardiomyocytes are shown in the bottom panel. (**c**) Immunofluorescence images ( × 400) showing the expression of ARC in cardiomyocytes in the control, HG, HG+mir-30d, and HG+AMO-mir-30d groups. Blue: nuclear staining (DAPI); green: ARC staining. (**d**) Relative protein and mRNA levels of caspase-1 in the control, mir-30d and NC groups. Caspase-1 levels in the HG, HG+AMO-mir-30d and HG+AMO-NC groups in high-glucose-treated cardiomyocytes are shown in the bottom panel. (**e**) Immunofluorescence images ( × 400) showing expression of caspase-1 in the control, HG, HG+mir-30d, and HG+AMO-mir-30d groups. Blue: nuclear staining (DAPI); green: caspase-1 staining. *n*=3. *P<0.05 and **P<0.01 *versus* control; ^#^*P*<0.05 and ^##^*P*<0.01 *versus* mir-30d; ^+^*P*<0.05 and ^++^*P*<0.01 *versus* HG; ^§^*P* <0.05 and ^§§^*P*<0.01 *versus* HG+AMO-mir-30d; mean±S.E.M.

ARC, an antiapoptotic gene, is known to be a transcriptional target of foxo3a;^31^ we therefore assessed the effect of mir-30d on the expression of ARC. Treatment of cardiomyocytes with high glucose (30 and 50 mmol/l) significantly inhibited ARC at both protein and mRNA levels (Figure 5a). Moreover, mir-30d overexpression mimicked high-glucose-induced downregulation of ARC in cardiomyocytes under normal glycemic environment, and knockdown of mir-30d restored ARC levels in cardiomyocytes under hyperglycemic conditions, as verified by western blotting analysis, real-time PCR methods and immunofluorescence staining (Figures 5b and c).

### Mir-30d regulates cardiomyocyte pyroptosis through foxo3a and ARC

Next, we attempted to elucidate the possible mechanisms through which mir-30d promotes cardiomyocyte pyroptosis. We found that transient expression of mir-30d activated caspase-1, as evidenced by increases in the caspase-1 mRNA and protein levels. On the other hand, caspase-1 level was elevated in cardiomyocytes seating in a high-glucose environment, and this elevation was abolished by transfection with AMO-mir-30d, indicating that knockdown of mir-30d attenuated caspase-1 expression (Figures 5d and e).

**Figure 6:**
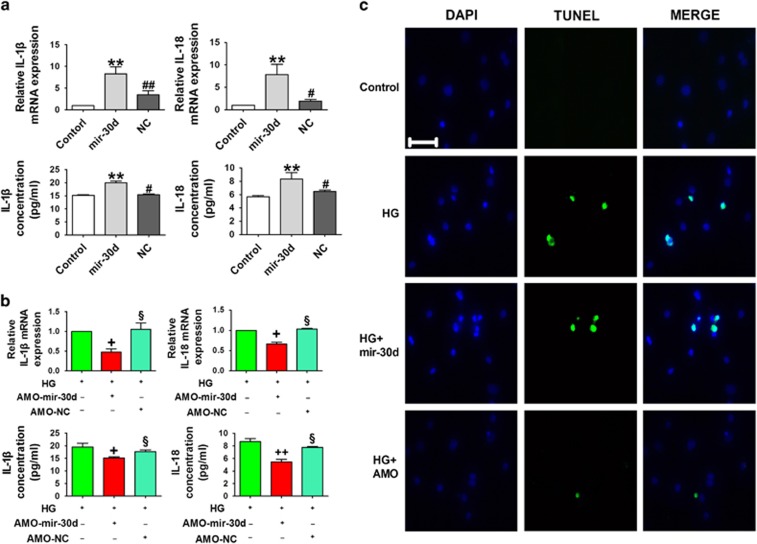
Mir-30d promotes cardiomyocyte pyroptosis. (**a**) Concentrations of IL-1*β* and IL-18 in the serum and mRNA levels in the hearts of the control, mir-30d and NC groups. (**b**) Concentrations of IL-1*β* and IL-18 in the serum and mRNA levels in the HG, HG+AMO-mir-30d and HG+AMO-NC groups. (**c**) TUNEL (terminal deoxinucleotidyl transferase-mediated dUTP-fluorescein nick end labeling) images showing cell death in the control, HG, HG+ mir-30d and HG+AMO-mir-30d groups. Blue, nuclear staining (DAPI); green, TUNEL staining; TUNEL nuclear localization in merged image. Scale bar: 20 *μ*m. *n*=3. *P<0.05 and **P<0.01 *versus* control; ^#^*P*<0.05 and ^##^*P*<0.01 *versus* mir-30d; ^+^*P*<0.05 and ^++^*P*<0.01 *versus* HG; ^§^*P*<0.05 *versus* HG+AMO-mir-30d; mean±S.E.M.

Moreover, to exploit the potential role of mir-30d in cardiomyocyte pyroptosis, we evaluated the effects of mir-30d on the levels of pro-inflammatory factors IL-1*β* and IL-18 using enzyme-linked immunosorbent assay (ELISA) and real-time PCR methods. Forced expression of mir-30d significantly increased IL-1*β* and IL-18 levels, which were partially diminished after transfection with AMO-mir-30d in high-glucose-treated cardiomyocytes (Figures 6a and b). TUNEL staining further confirmed that transfection of mir-30d exacerbated high-glucose-induced cell death, which was predictably reversed by AMO-mir-30d (Figure 6c).

**Figure 7:**
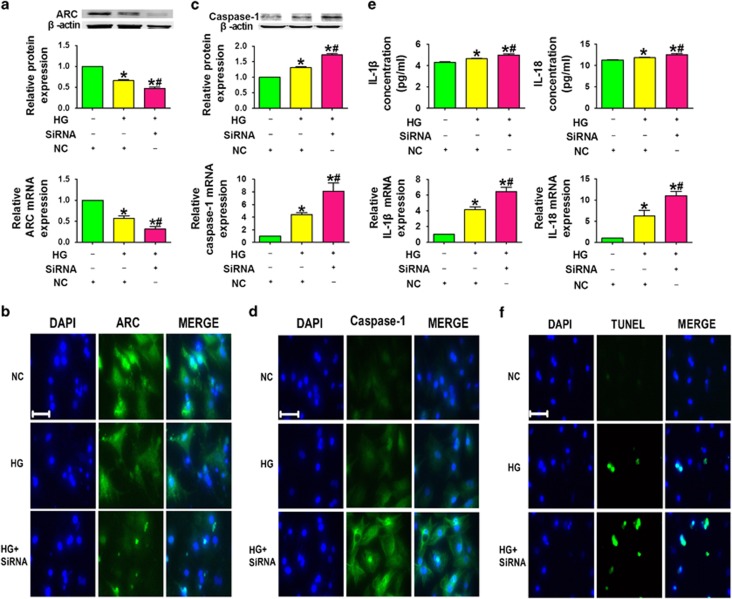
The effect of mir-30d on pyroptosis is mediated through downregulation of ARC. (**a**) ARC levels in the NC, HG+NC and HG+siRNA groups. (**b**) Immunofluorescence images ( × 400) showing the expression of ARC in cardiomyocytes in the NC, HG+NC and HG+siRNA groups. Blue: nuclear staining (DAPI); green: ARC staining. (**c**) Caspase-1 protein and mRNA levels in the NC, HG+NC and HG+siRNA groups. (**d**) Immunofluorescence images ( × 400) showing the expression of caspase-1 in cardiomyocytes in the NC, HG+NC and HG+siRNA groups. Blue: nuclear staining (DAPI); green: caspase-1 staining. (**e**) IL-1*β* and IL-18 levels in the NC, HG+NC and HG+siRNA groups. (**f**) TUNEL (terminal deoxinucleotidyl transferase-mediated dUTP-fluorescein nick end labeling) images showing cell death in the three groups. Blue, nuclear staining (DAPI); green, TUNEL staining; TUNEL nuclear localization in merged image. *n*=3. *P<0.05 *versus* NC; ^#^*P*<0.05 *versus* HG+NC; mean±S.E.M.

It was likely that mir-30d exerted its effect via downregulating ARC. To test this notion, we transfected ARC siRNA into cardiomyocytes under high-glucose conditions. We observed a pronounced elevation of caspase-1 expression. Additionally, ARC knockdown significantly increased caspase-1, IL-1*β* and IL-18 levels. Gene-specific inhibition of ARC exacerbated high-glucose-induced pyroptosis (Figure 7).

## Discussion

**Figure 8:**
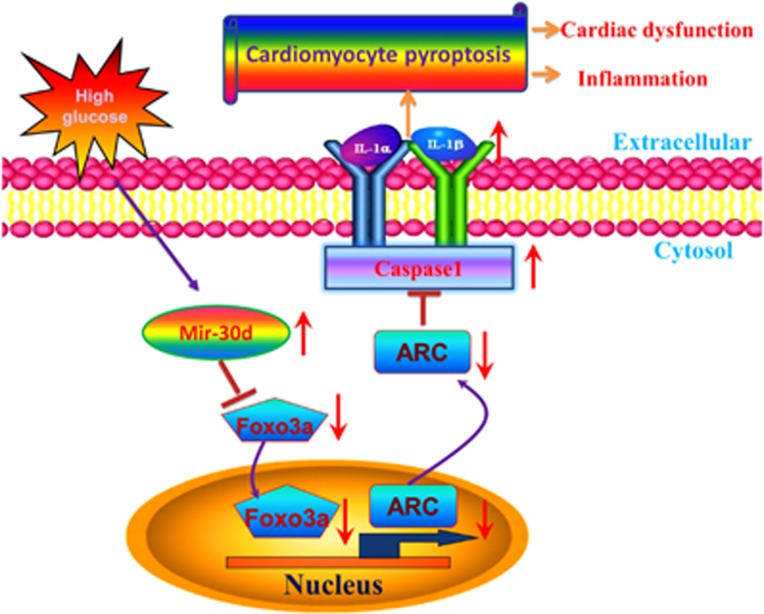
Schematic diagram for the proposed mir-30d–pyroptosis signaling pathways. Mir-30d is significantly elevated in a high glucose condition and has a regulatory role in pyroptosis by downregulating foxo3a and the downstream protein ARC, leading to upregulation of caspase-1 expression and pro-inflammatory cytokines IL-1*β* and IL-18 in diabetic cardiomyopathy

In the present study, we unraveled a novel role of mir-30d in diabetic cardiomyopathy. Mir-30d was upregulated in high-glucose-treated cardiomyocytes and in the diabetic hearts as well. Mir-30d repressed foxo3a and ARC expression, upregulated expression of inflammatory molecules and promoted pyroptosis *in vitro* and *in vivo*. Knockdown of mir-30d by its antisense inhibitor AMO-mir-30d markedly diminished the effects of mir-30d, as summarized in Figure 8.

In DM, high blood glucose is an independent causal factor for cardiomyopathy, one of the leading causes of hospitalization and death worldwide.^32^ Diabetic cardiomyopathy contributes to the inability of an abnormally enlarged, thickened and stiffened heart to pump blood effectively. Several hypotheses have been proposed to explain the pathogenesis of diabetic cardiomyopathy: (1) cardiac metabolic disturbances are important contributors to the development of diabetic cardiomyopathy;^33^ (2) oxidative stress and reactive oxygen species accumulation are associated with diabetic cardiac complications;^34^ (3) calcium signaling is a major regulator of contractile function in the diabetic heart;^35^ (4) functional and structural alterations in mitochondria contribute to diabetic cardiac dysfunction;^36^ and (5) diabetic cardiomyopathy is a multi-organ inflammatory disease and is associated with chronic low-grade inflammation in the heart,^37^ which has an important role in the progression of cardiac fibrosis.^38^

Pyroptosis is a highly inflammatory form of programmed cell death and is triggered by caspase-1 activation.^16,39,40^ In recent years, a tremendous amount of efforts has been devoted to understanding the mechanisms of pyroptosis in many diseases and to determine the genes and pathways involved in this process. Zheng *et al.*^41^ reported that pyroptosis is a catastrophic form of cell death in cardiovascular diseases. Recent studies have shown that blocking pyroptosis was effective in either slowing or reducing cell injury in models of heart disease.^39^ However, the molecular components regulating pyroptosis in diabetic cardiomyopathy remain largely unknown. In our study, we successfully produced STZ-induced diabetic cardiomyopathy models, which demonstrated decreased systolic and diastolic function, interstitial fibrosis and myocardial hypertrophy, as shown in Figure 1 and Supplementary Table S1. Diabetic rats and cardiomyocytes when exposed to a high-glucose environment displayed increased levels of caspase-1, IL-1*β* and IL-18. The results suggested that pyroptosis was induced in both the animal model of diabetic cardiomyopathy and cardiomyocytes treated with high-glucose to mimic hyperglycemia. We further elucidated the molecular mechanisms involved in these processes.

Recently, miRNAs have emerged as important mediators of translational control and as regulators of a wide range of biological processes.^17^ Overexpression of miRNAs is a common phenomenon that occurs in various diseases, and aberrantly expressed miRNAs often participate in the pathogenesis of specific diseases, including diabetes. For example, mir-133 and mir-30d have been documented to directly downregulate connective tissue growth factor and may be potential therapeutic strategies for the prevention of the progression of structural changes in the extracellular matrix of myocardial cells.^42,43^ Pan *et al.*^44^ reported that circulating mir-30d may be an important marker for diagnosis of hypertrophy. Furthermore, several studies showed that mir-30d was significantly upregulated under high-glucose conditions.^45,46^ However, there is no direct evidence indicating the role of mir-30d in pyroptosis in the setting of diabetic cardiomyopathy or the mechanisms underlying this disease process. In this study, we identified several miRNAs that are highly relevant to diabetic cardiomyopathy and found that mir-30d expression was remarkably increased both in high-glucose-treated cardiomyocytes and in heart tissues of diabetic rats. Foxo3a is involved in the inhibition of cell death and promotion of cellular growth and in cardiac remodeling in diabetes.^47^ Several miRNA databases, such as TargetScan, miRDB and miRanda, show that foxo3a is a target gene for mir-30d, which is conserved among different species. In this study, we used both gain- and loss-of-function techniques to manipulate mir-30d expression to investigate its regulatory effects on foxo3a in cardiomyocytes. Luciferase reporter gene assay as well as protein and mRNA expression detections validated that foxo3a is a direct target of mir-30d. More importantly, overexpression of mir-30d resulted in increased levels of caspase-1, IL-1*β* and IL-18, whereas knockdown of mir-30d attenuated cardiomyocyte inflammatory cell death, indicating anti-mir-30d as a strategy for the prevention of cardiac pyroptosis. To go further, we determined that mir-30d may affect myocardial pyroptosis via targeting foxo3a and its downstream protein, ARC.

Zhang *et al.*^48^ reported that ARC protects rat cardiomyocytes against oxidative stress through inhibition of a caspase-2-mediated mitochondrial pathway. Another study showed that ARC is critical to cardiomyocyte survival in the setting of doxorubicin cardiotoxicity.^49^ Chatterjee *et al.*^50^ demonstrated that ARC gene transfected by adenovirus into rabbits with ischemia and reperfusion injury reduces apoptosis. Conversely, genetic ablation of ARC accelerates cardiomyopathy in the setting of ischemia–reperfusion injury.^51^ Lu *et al.*^31^ further revealed that ARC is a transcriptional target of foxo3a in cardiomyocytes. On the basis of these findings, we hypothesized that suppression of ARC is a mechanism through which mir-30d affects pyroptosis. Indeed, ARC silencing by siRNA resulted in cardiomyocyte pyroptosis, suggesting that ARC functions to suppress pyroptosis in diabetic cardiomyopathy. Our results therefore revealed a novel mechanism for cardiac pyroptosis regualtion.

Taken together, our results provide the first evidence that mir-30d is dysregulated in diabetes and that it directly targets foxo3a. These findings unraveled a heretofore unknown pathway composed of mir-30d, foxo3a, ARC and caspase-1 that regulates myocardial pyroptosis. However, it is undeniable that the effect of mir-30d on diabetic cardiomyopathy may be mediated by a variety of mechanisms in addition to the foxo3a–ARC–caspase-1 pathway, and further investigation is required to unveil the other mechanisms involved. The salient findings from the present study indicate the essential role of mir-30d in diabetic cardiomyopathy and will greatly improve our understanding of the role of pyroptosis in diabetic cardiomyopathy and may provide an effective therapeutic approach for the conditions associated with pyroptosis.

## Materials and Methods

### Ethics statement

The study was approved by the ethics committee of Harbin Medical University, and all experimental procedures were approved by the Animal Care and Use Committee of Harbin Medical University. Our study was performed in accordance with the recommendations of the Guide for the Care and Use of Laboratory Animals, published by the US National Institutes of Health (NIH Publication no. 85-23, revised 1996).

### Animals and establishment of diabetic model

Wistar rats (180–220 g) were housed at an ambient temperature of 22±1 °C and a humidity of 55±5%, with food and water available *ad libium*. The rats were divided randomly into two groups: the control group and the diabetic model (DM) group. Rats in the DM group were gavaged with a high-fat diet (2 ml/day) prepared with lard (20%), cholesterol (5%), sucrose (5%), glucose (5%) and salt (6%) and emulsified in 20% Tween 80 and 30% propylene glycol with distilled water. Diabetic rats were injected intraperitoneally with 35 mg/kg/d of STZ (Sigma, St. Louis, MO, USA) dissolved in 0.1 M citrate buffer solution (pH=4.3), for 3 days. Fasting blood glucose levels were measured 72 h after STZ injection (ACCU-CHEK Active, Roche, Mannheim, Germany), and diabetes was considered successfully established in rats with blood glucose levels >16.7 mmol/l.^52^

### Echocardiography

Transthoracic echocardiography was performed using an ultrasound machine (Vivid 7, GE Medical, Milwaukee, WI, USA) equipped with a 10-MHz phased array transducer. LV internal dimensions (LVID; s) (LVID; d), interventricular septum thickness (IVS; s and IVS; d) and LV posterior wall thickness (LVPW; s and LVPW; d) were each measured during systole and diastole. EF % and FS % were calculated. M-mode recordings were performed at the level of the papillary muscles according to the double-blind procedure.^4^

### Biochemical estimations

Blood samples were collected from tail veins to determine fasting blood glucose levels. TC, TG, LDL and HDL were measured using kits according to the manufacturer's instructions (Nanjing Jiancheng Bioengineering Institute, Nanjing, China; Beijing Beihuakangtai Clinical Reagent Ltd., Beijing, China and Zhejiang Dongou Diagnostic Products Co., Wenzhou, China).

### HE and Masson's trichrome staining

Tissues were fixed in 4% paraformaldehyde. The samples were embedded in paraffin, cut into 5-*μ*m thick sections and stained with HE and Masson trichrome for histological and collagen analysis.

### Transmission electron microscopy

Selected samples were fixed in 2.5% glutaraldehyde in 0.1 mol/l phosphate-buffered saline (PBS) (pH=7.35) and subsequently rinsed in buffer, post-fixed in PBS 1% OsO4 for 2 h, stained en bloc with 1% uranyl acetate, dehydrated in graded ethanol and embedded in epoxy resin by routine methods. The sections were electron-stained and observed under a JEM-1200 electron microscope (JEOL Ltd., Tokyo, Japan).^53^

### Primary isolation and culture of cardiomyocytes

Primary cultures of neonatal cardiomyocytes were taken from the hearts of 1- to 3-day-old Wistar rats via collagenase digestion as described previously. Briefly, cardiac tissues were digested by pancreatin, and the isolated cells were resuspended in Dulbecco's modified Eagle's medium (Hyclone, Logan, UT, USA) containing 10% fetal bovine serum (Hyclone). Cardiomyocytes were purified by differential plating, and 0.1 mM 5-bromo-2-deoxyuridine was added to the medium to deplete non-myocytes. Cardiomyocytes were incubated at 37 °C in humidified air with 5% CO_2_.^54^

### Gene transfection

The mir-30d mimic, negative control (NC) miRNA, AMO-mir-30d and AMO-NC were synthesized by Guangzhou Ribo Bio Co., Ltd. (Guangzhou, China). Cells were transfected using X-treme GENE siRNA Transfection Reagent (catalog no. 04476093001; Roche, Mannheim, Germany) according to the manufacturer's instructions. The siRNA-targeting rat ARC and the siRNA non-targeting the NC were each designed and synthesized by GenePharma (Shanghai, China). The ARC siRNA sequence is 5′-CGGAAACGGCUGGUAGAAATT-3′ and the antisense sequence is 5′-UUUCUACCAGCCGUUUCCGTT-3′. Measurements were performed 24 h after the transfection.

### Enzyme-linked immunosorbent assay

Arterial blood serum was collected for measurement of IL-1*β* and IL-18 concentrations using an ELISA kit according to the manufacturer's instructions (uscn-SEA064R and uscn-SEA563Ra).^55^

### RNA extraction and real-time PCR

The animals were euthanized, and the hearts were dissected and frozen in liquid nitrogen. Total RNAs from cultured neonatal cardiomyocytes or heart tissues were extracted using 1 ml of Trizol reagent (Invitrogen, Carlsbad, CA, USA) according to the manufacturer's instructions. CDNA synthesis was performed using the High Capacity cDNA Reverse Transcription Kit (Applied Biosystems, Carlsbad, CA, USA, Cat. no. 4368814) according to the manufacturer's instructions. The SYBR Green PCR Master Mix Kit (Applied Biosystems, Cat. no. 4309155) was used to quantify the relative mRNA levels of mir-30d, foxo3a, ARC, caspase-1, IL-18 and IL-1*β*. Real-time PCR was performed with the 7500 FAST Real-Time PCR System (Applied Biosystems) for 40 cycles, with GAPDH and U6 serving as internal controls.^56^ The following primers were used in the study:

Foxo3a: Forward, 5′-TGCCGATGGGTTGGATTT-3′,

Reverse, 5′-CCAGTGAAGTTCCCCACGTT-3′

ARC: Forward, 5′-ATGGGTAACATGCAGGAGCGC-3′,

Reverse, 5′-GTCCAGCAGCAACCCAGAGTC-3′

Caspase-1: Forward, 5′-ACACGTCTTGCCCTCATTATCT-3′,

Reverse, 5′-ATAACCTTGGGCTTGTCTTTCA-3′

IL-1*β*: Forward, 5′-CCCTGCAGCTGGAGAGTGTGG-3′,

Reverse, 5′-TGTGCTCTGCTTGAGAGGTGCT-3′ and

IL-18: Forward, 5′-ACAACCGCAGTAATACGGAGCA-3′,

Reverse, 5′-TGTGCTCTGCTTGAGAGGTGCT-3′.

### Western blotting

Protein samples of 100 *μ*g each were loaded on a 10% SDS-polyacrylamide gel and transferred onto a nitrocellulose filter membrane, which was subsequently blocked by 5% non-fat milk dissolved in PBS for 2 h. The blots were probed with primary antibodies against caspase-1 (Cell Signaling Technology, Danvers, MA, USA), foxo3a (Abcam, Cambridge, MA, USA) and ARC (Proteintech Group Inc., Chicago, IL, USA). *β*-Actin (Santa Cruz Biotechnology, Inc., Dallas, TX, USA) was used as an internal control. Western blotting bands were quantified using the Odyssey Infrared Imaging System (LI-COR, Lincoln, NE, USA) by measuring band intensity (Area × OD).

### Immunofluorescence staining and immunocytochemistry

For immunofluorescence staining, cultured neonatal cardiomyocytes were fixed with 4% buffered paraformaldehyde in PBS. Blocking solution (1% BSA and 0.1% Triton-X in PBS) was used to penetrate and incubate fixed cells at room temperature for 2 h. Primary antibodies against foxo3a, caspase-1 and ARC were placed in PBS overnight at 4 °C, followed by incubation with the appropriate secondary antibody (Invitrogen) for 1 h at room temperature. The nuclei were stained using 4',6-diamidino-2-phenylindole (DAPI; Beyotime, Shanghai, China) for 20 min at room temperature. Immunofluorescence was examined under a fluorescence microscope (Nikon 80i, Otawara, Tochigi, Japan).

For immunohistochemical analysis, frozen heart section specimens were fixed with 4% buffered paraformaldehyde embedded in paraffin. Specimens were dehydrated by an ascending series of ethanol and cleared with xylene. All sections were immunostained with primary antibodies against caspase-1, foxo3a or ARC at 4 °C overnight. After incubation with secondary antibodies, the sections were stained with diaminobenzidine.^15,57^

### Luciferase reporter assays

Both foxo3a 3′-UTRs containing conserved mir-30d-binding sites and the mutated 3′-UTRs were synthesized by Sangon Biotech (Shanghai) Co., Ltd. (Shanghai, China), and amplified by PCR. The forward primer was 5′-CCGCTCGAGAGGATCACTGAGGAAGGGGAAGTG-3′ and the reverse primer was 5′-ATAAGAATGCGGCCGCGCCTTGTACTACACATGTGTGACTGATC-3′. The PCR fragment was subcloned into the XhoI and NotI sites downstream from the luciferase gene in the psi-CHECK2 vector (Promega Biotech Co., Ltd., Madison, WI, USA). The 3′-UTR luciferase vector (100 ng) was cotransfected with either mir-30d mimics or AMO-mir-30d into human embryonic kidney 293 (HEK293) cells using Lipofectamine 2000 (Invitrogen), with 10 ng of Renilla luciferase reporters used as an internal control. After 48 h, the cells were collected and lysed. A luciferase activity assay was performed using the Dual-Luciferase Reporter Assay System (Promega Biotech Co., Ltd.) according to the manufacturer's instructions.^58^

### TUNEL staining

Cells were plated onto coverslips in 24-well culture plates, and *In Situ* Cell Death Detection Kits (TUNEL fluorescence FITC kit, Roche, Indianapolis, IN, USA) were used to detect DNA fragmentation of individual cells according to the manufacturer's instructions. The nuclei were stained with DAPI. TUNEL staining was assessed via fluorescence microscopy (Eclipse 80i; Nikon Co., Tokyo, Japan).^59^ Nuclei that were double labeled with DAPI and TUNEL were considered positive.

### Statistical analysis

Continuous variables are presented as mean±S.E.M. One-way ANOVA was carried out for multiple comparisons by GraphPad Prism 5.0 (GraphPad Software, Inc., La Jolla, CA, USA). *P*-values <0.05 indicated a statistically significant difference.

## Abbreviations

ARC: apoptosis repressor with caspase recruitment domain
DAPI: 4',6-diamidino-2-phenylindole
DM: diabetes mellitus
ELISA: enzyme-linked immunosorbent assay
Foxo3a: Forkhead box O3
HDL: high-density lipoprotein
IL: interleukin
LDL: low-density lipoprotein
miRNA: microRNA
PBS: phosphate-buffered saline
STZ: streptozotocin
TC: total cholesterol
TG: triglyceride
NC: negative control
