# Supplementary material for: MicroRNA-30d regulates cardiomyocyte pyroptosis by directly targeting foxo3a in diabetic cardiomyopathy
Source: Cell Death Dis. 2014 Oct 23;5(10):e1479–. doi: 10.1038/cddis.2014.430 (PMC4237254; doi:10.1038/cddis.2014.430)
Supplement: Supplementary Table S1 [file cddis2014430x1.doc]

**Supplementary Tables and Table Legends**

**Supplementary Table S1: Levels of general characteristics in the rats**

Parameters in the Control and DM groups indicating the successfully established diabetic rats. *p＜0.05 vs Control.

| Parameter | Control | Diabetic model |
| --- | --- | --- |
| Food intake | 20.9±0.1 | 22.3±0.2 |
| Water intake | 68.2±1.0 | 122.0±0.4* |
| Body weight | 288.2±8.6 | 226.1±3.2* |
| Blood glucose | 7.0±0.2 | 16.2±0.3* |
| TCH | 3.4±0.1 | 10.0±0.4* |
| TG | 0.8±0.1 | 5.2±0.6* |
| LDL  HDL | 1.7±0.1  1.0±0.1 | 4.2±0.1*  0.6±0.1* |
